# Tai Chi Quan Versus Physical Therapy on Pain and Cognitive Performance for Elderly People With Chronic Low Back Pain: Study Protocol for a Randomized Controlled Trial

**DOI:** 10.3389/fnagi.2022.900430

**Published:** 2022-06-16

**Authors:** Rui Wang, Dong Zhu, Lin Wang, Jing Liu, Jun Zou, Yang Sun, Yan Jiang, Hao-Yu Hu, Zhi-Wei Deng, Lin-Man Weng, Kang-Yong Zheng, Suparata Kiartivich, Xue-Qiang Wang

**Affiliations:** ^1^Department of Sport Rehabilitation, Shanghai University of Sport, Shanghai, China; ^2^School of International Education, Shanghai University of Sport, Shanghai, China; ^3^Department of Rehabilitation Medicine, Shanghai Shangti Orthopaedic Hospital, Shanghai, China; ^4^Department of Martial Arts, Shanghai University of Sport, Shanghai, China

**Keywords:** chronic low back pain, traditional Chinese exercise, physical therapy, cognition, randomized controlled trial

## Abstract

**Objectives:**

Chronic low back pain has become a major cause of global disability and caused a huge economic burden to society. Physical therapy is a vital strategy for rehabilitation of chronic low back pain. Although several trials have shown that Tai Chi Quan is a beneficial treatment, the comparative effectiveness of Tai Chi Quan versus physical therapy is unknown. We are conducting a randomized controlled trial to assess the effectiveness of Tai Chi Quan versus that of physical therapy in treating chronic low back pain.

**Methods:**

We will perform a single-blind randomized controlled trial on elderly people with chronic low back pain. 138 participants will be randomly assigned to the Tai Chi Quan group (60-min classes, three times per week for 12 weeks) or physical therapy group (10 min of evaluation and warm-up, 40 min of therapist-directed exercise therapy, and 10 min of relaxation, three times per week for 12 weeks) with an allocation of 1:1. The participants will be followed up for 40 weeks for the study of long-term effects. The primary outcomes include pain intensity and back-related function at 12 weeks. Secondary outcomes include lumbar quantitative sensory testing, balance, cognitive function, psychosocial function, cost-effectiveness, compliance and adverse events. We will perform the intention-to-treat analysis for withdrawal and missing data.

**Discussion:**

The study will be the first randomized trial with comparative-effectiveness of Tai Chi Quan and physical therapy for chronic low back pain. Standardized protocol, large sample size, and comprehensive outcomes are important features in this trial. This study aims to determine the feasibility and effectiveness of Tai Chi Quan for low back pain. The results of this study will be beneficial for elderly people with low back pain and medical rehabilitation personnel.

**Clinical Trial Registration:**

www.chictr.org.cn, identifier ChiCTR2000029723.

## Introduction

Low back pain (LBP) is a common pain syndrome between the lower rib margins and buttock creases and occurs at all ages (Hartvigsen et al., 2018). When LBP lasts more than 3 months, it is called chronic low back pain (CLBP), which is more common in women and the elderly (Hoy et al., 2012). By 2017, LBP has prevailed as one of the leading causes of global years lived with disability (YLD) and non-fatal health loss (GBD 2015 Disease and Injury Incidence and Prevalence Collaborators, 2018), and YLD increased by 54% between 1990 and 2015, especially in low- and middle-income countries (GBD 2015 Disease and Injury Incidence and Prevalence Collaborators, 2016). The prevalence of LBP is more than 80%, of which CLBP accounts for 23%, and the disability rate is 11–12% (Brinjikji et al., 2015; Maher et al., 2017). The prevalence of LBP ranged from approximately 28 to 51% in 28 countries (Maher et al., 2017). A systematic review of 35 studies found that the prevalence of LBP ranged from 21 to 75% among approximately 130,000 people aged over 60 years, and the majority of patients had functional disabilities and limitations of physical capacity (de Souza et al., 2019). Owing to changes in social environment and lifestyle, physical inactivity and sedentarism have become worldwide issues and important predictors of poor health (Panahi and Tremblay, 2018), and the incidence of adolescents with LBP shows an increasing trend (Shim et al., 2014). People with CLBP suffer from unpleasant nociceptive sensation and limited activities, are often absent from work and have low quality of life (Deyo et al., 2014). The cost of LBP ranked sixth in the overall burden of diseases (“Global, regional, and national incidence, prevalence, and years lived with disability for 301 acute and chronic diseases and injuries in 188 countries, 1990–2013: a systematic analysis for the Global Burden of Disease Study 2013 Collaborators, 2015). Studies have pointed that LBP forces older people to leave work and retire early; this situation results in huge indirect costs (mainly lost productivity and household income) (Ferreira et al., 2010). People who retire early because of LBP earn approximately 87% less than people who work full time (Schofield et al., 2011). In 2018, the World Health Organization has identified research on ‘reducing disabling LBP’ as one of its global health priority programs (Buchbinder et al., 2018). LBP is of urgent global public health concern.

The non-invasive management for CLBP usually includes medication, physical therapy (PT) and education about being physically active (Qaseem et al., 2017). PT consists of movement therapy (such as aerobic exercise, whole body vibration exercise, and core stability exercise), manual therapy (such as massage, spinal manipulation, spinal mobilization, and nerve mobilization) and modality therapy (such as electrotherapies, laser therapy, and superficial heat therapy), and the international guidelines for LBP have provided a specific set of recommendations for these three sections (de Campos, 2017; Qaseem et al., 2017; George et al., 2021). Movement therapy has been recognized as a core part of LBP treatment (R. Wang et al., 2020). A systematic review have shown that guided and personalized exercise programs can effectively improve CLBP (Hayden et al., 2005). In clinical randomized controlled trials (RCTs), PT is often compared with other LBP interventions, and cost-benefit analyses are performed. Saper et al. founded that yoga and PT are effective for CLBP after 12-week intervention, and yoga is not inferior to PT in terms of improving lumbar function and relieving pain (Saper et al., 2017). Vibe et al. compared the effects of cognitive function therapy with those of PT for CLBP and showed that PT fully reflects the emphasis on exercise therapy and the importance of classified diagnosis and treatment (Vibe Fersum et al., 2013). Pengel et al. used physiotherapist-directed exercise interventions in patients with LBP, and their pain and physical function scores were significantly better than those of the placebo group after 6 weeks (Pengel et al., 2007). PT plays an important role in musculoskeletal disorders. The national PT referral rate for LBP patients aged 45 to 59 years in the United States between 1997 and 2010 was estimated to be about 36.5%, and the rate was approximately 19% for LBP patients aged 60 and above (Zheng et al., 2017). In the Chinese clinical setting, patients with chronic pain are mostly managed by rehabilitation therapists, and those with high incomes are particularly likely to choose individualized therapists. Although therapist-directed PT is effective in resolving the problems of patients, the cost of PT is considerable for most low- and middle-income people with CLBP. At present, health care staff and patients are looking forward to a long-term effective alternative treatment with lower economic burden.

As a traditional exercise therapy, Tai Chi Quan (TCQ) has been widely used in researching health promotion interventions for posture balance and fall and chronic diseases (Liu et al., 2012; X. Wang et al., 2015; Zhi-lei and Dong, 2018). A systematic review has demonstrated the safety and reliability of the clinical application of TCQ (Wayne et al., 2014). In 2017, the clinical guideline issued by the American College of Physicians strongly recommended TCQ for CLBP (Qaseem et al., 2017). TCQ is considered an active therapy and a coordinated movement in which a practitioner needs to concentrate and coordinate breathing. TCQ involves circular and curved movements, emphasizes the twisting of the waist and hips and drives the limbs into diagonal spiral movements. The forward and backward, left and right and oblique steps can improve the flexibility and stability of the joints (Li, 2014). In recent years, some important achievements about TCQ have been made in health promotion and disease intervention (Li et al., 2012, 2018; C. Wang et al., 2016). In 2011, an RCT of TCQ for persistent LBP found that 10 weeks of TCQ exercise improves pain levels and pain-related disability (Hall et al., 2011). In 2013, Another RCT showed that 6 months of TCQ is effective in reducing pain symptoms and can be used as an alternative therapy (Sun, 2013).

Tai Chi Quan can significantly improve core stability and muscle strength, thereby reducing the risk of falls in patients with CLBP (Lomas-Vega et al., 2017). Sleep problems are common among people with CLBP, and insomnia can increase the risk of CLBP (Skarpsno et al., 2020). A meta-analysis showed that TCQ has a significant beneficial effect on the sleep quality of the elderly (Du et al., 2015). The intensity of TCQ practice depends on time, speed and experience. For the elderly with CLBP, simplified TCQ is easy to learn and can improve balance, coordination and spinal stability. Compared with the waiting group and the usual care group, TCQ from 10 to 28 weeks had a better effect on lumbago (Kong et al., 2016). However, few studies have investigated the long-term follow-up effects of TCQ on CLBP. Although PT is the main management method for LBP rehabilitation, it is expensive in terms of manpower and financial resources. As an easy to learn and low- cost exercise therapy, TCQ is expected to be accepted as an alternative therapy by a large number of patients with CLBP. However, previous studies had some deficiencies, such as short intervention time, lack of follow-up observation and economic analysis, and evidence supporting the application value of TCQ in patients with CLBP remains insufficient. Therefore, we propose a detailed protocol to study the comparative effectiveness of TCQ classes versus grouped-PT for people with CLBP. Our primary hypothesis is that TCQ and grouped-PT have a significant clinical effect on CLBP. The secondary hypothesis is that TCQ is not inferior to PT in terms of improving outcomes. The third hypothesis is that TCQ has a longer-term effect than PT.

## Study Design and Methods

### Study Design

**FIGURE 1:**
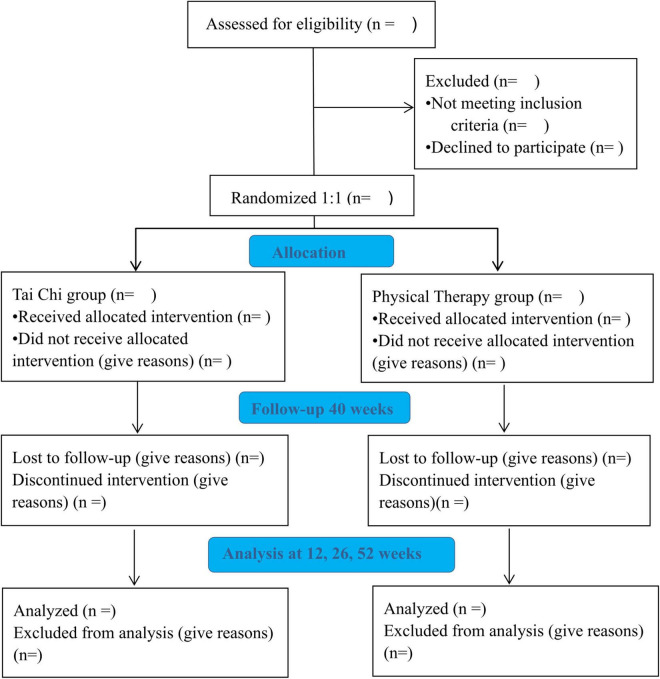
Study flow diagram.

The study is a single-blinded RCT of TCQ classes or grouped-PT for patients with CLBP (Figure 1. Study flow diagram). Eligible participants will be randomly assigned into the Tai Chi Quan group (TCQG) or physical therapy group (PTG). Both groups will perform exercises for 60 min three times a week for a total of 12 weeks. After the program, 40 weeks of follow-ups will be conducted.

The coprimary outcomes after the interventions are maximum and average pain intensity measured using a numerical rating scale (NRS) (Williamson and Hoggart, 2005) and back-related function measured using the Roland Morris Disability questionnaire (RMDQ) (Roland and Morris, 1983). The secondary outcomes include lumbar quantitative sensory testing, balance, cognitive function, psychosocial function, cost-effectiveness, compliance and adverse events. Healthcare utilization data, including medical costs, drugs use and cost of TCQ and PT, are tracked to estimate whether TCQ would be more economical and beneficial than grouped-PT exercise. The outcomes and measurement timepoints are presented in Table 1.

**TABLE 1:** Measurements timing for primary and secondary outcomes.

Outcomes variables	Baseline	Week 12	Week 26 (follow-up)	Week 52 (follow-up)
**Primary outcomes**				
NRS – the Maximum Pain	√	√	√	√
NRS – the Average Pain	√	√	√	√
RMDQ – Back-related Function	√	√	√	√
**Secondary outcomes**				
Lumbar quantitative sensory testing	√	√		
4-stage balance test				
Trial making tests	√	√	√	√
Stroop test	√	√	√	√
6-min walk test	√	√	√	√
Geriatric depression scale	√	√	√	√
Self-rating anxiety scale	√	√	√	√
Pain catastrophizing scale	√	√	√	√
Tampa scale for Kinesiophobia	√	√	√	√
Pittsburgh sleep quality index	√	√	√	√
Short-form 12	√	√	√	√
General perceived self-efficacy scale	√	√	√	√
Expenses for healthcare monthly	√	√	√	√
Expense for other diseases	√	√	√	√
Expense for low back pain	√	√	√	√
Global perceived effect		√		
Adherence		√		
Adverse Events		√		

*NRS, numerical rating scale; RMDQ, Roland Morris disability questionnaire.*

The study setting is located in the Center of Sports Medicine and Rehabilitation, Shanghai Shangti Orthopaedic Hospital (SSOH), Shanghai, China. Each participant will sign a written informed consent. This study protocol has been approved by the Ethics Committee of the Shanghai University of Sport, China (Grant number 102772019RT039).

### Sample Size

The sample size analysis is based on hypothesized change in RMDQ scores. For the primary outcome of change in pain intensity from baseline to week 12, we assume that two-sided α error prob is equal to 0.05 and power is 0.8. The minimal clinically important difference of the RMDQ is prespecified for 3 points (Jordan et al., 2006), and the effect size of RMDQ is in the range of 0.54–0.84 (Saper et al., 2017). We used G-Power Software (version 3.1.9.2, Germany) to estimate the total sample size with the method of *t*-tests-means (difference between two independent means measures in two groups). Considering the effect size of RMDQ is 0.54 and 20% attrition rate, we need a minimum of 138 participants in this study or 69 participants in each group to detect whether 12 weeks of TCQ and grouped-PT have a significant clinical effect on CLBP.

### Participants

#### Randomization and Blinding

We will take a community randomized approach, and four communities will be assigned with an allocation of 1:1 through the random numbers generated by the analyst. Finally, two communities will be assigned to the TCQG, and two to the PTG. We will recruit participants in the corresponding community to practice TCQ or PT. Throughout the courses of the trial, all study assessors who are responsible for collecting outcomes will be blinded to the hypotheses and allocation. Meanwhile, we shall maintain separation between the assessors and assistants who are responsible for recruitment work and between the assessors and class instructors. In addition, participants should conceal their group status to the assessors. Participants have informed consent and are blinded impossibly without a placebo. Finally, all data analyst do not know group allocation and deal with coded data instead of the names of the participants.

#### Inclusion and Exclusion Criteria

Target participants will be mainly community-dwelling elderly people who are living in Shanghai. The specific inclusion and exclusion criteria are listed in Table 2. The participants will quit the study if they have been diagnosed with severe diseases (such as heart failure) or serious adverse events occur during the experiment. Besides, the participants will be allowed to voluntarily quit as result of any of their own reasons. We plan to execute the following ways to access our participants as many as possible: (1) Distributing study recruitment flyers in nearby four communities with permission; (2) Sending study recruitment presentations to community healthcare center staff to help introduce potential participants; and (3) Disseminating the recruitment information through electronic communications and anyone interested can contact the recruiter by telephone.

**TABLE 2:** Inclusion criteria and exclusion criteria.

Inclusion criteria	Exclusion criteria
Aged 50–80 years, can speak Chinese and complete the questionnaire survey.	Have practiced tai chi regularly for any reason within the past 6 months or who have practiced tai chi, yoga or qigong for nearly half a year to treat LBP.
Pain, at least 3 months, typically occurs in the area between the lower rib margins and the buttock creases, commonly accompanied by pain in one or both legs.	Have received guided physical therapy for LBP within half a year, including stretching, strength training, motor control training, and so on.
Have had back pain on at least half the time in the last 6 months.	Have specific causes or potential causes of LBP (e.g., sciatica, spinal stenosis, lumbar disk herniation, spondylolisthesis, recent vertebral fracture, and so on).
The average scores of numerical rating scale (in the range of 0–10) ≥3 and ≤8.	Have red flags of serious underlying systemic or visceral disease (e.g., inflammatory disorders malignancy, unexplained weight loss, infections, or recent trauma).
Back-related function scores of Roland-Morris disability questionnaire (in the range of 0–24) ≥5.	Pain in other parts of the body is greater than low back pain.
Have normal cognition or only mild cognitive impairment [the scores of Mini-Mental State Examination (in the range of 0–27) ≥24].	Had prior lumbar spine surgery.
Able to understand the learning process and complete the whole course.	Have history of drug and alcohol abuse.
Volunteer to participate the trial and sign an informed consent.	Have other disabling conditions that might confound treatment effects.
	Have moderate to severe depressive symptoms [the scores of Geriatric Depression Scale (in the range of 0–30) ≥21].
	Exercise is contraindicated.

### Intervention Conditions

#### Intervention Classes Locations

The participants randomized to TCQG will practice it at community health centers or residential activity center for non-profit organizations where the evidenced-based prevention of chronic diseases and health promotion activities are encouraged. PT intervention will take place at the Center of Sports Medicine and Rehabilitation of SSOH or community resident activity centers.

#### Tai Chi Quan Group

Tai Chi Quan is strongly recommended for treating CLBP (Qaseem et al., 2017). In our study, the TCQ program features eight modified movements, which combine Yang style and Cheng style, and reflect multiple directions around the central axis. In general, these movements revolve around the waist as the core, thereby activating the limbs. The movements include various steps (forward, backward and lateral), spatial orientation movements (performing each single movement in different directions) and complex movements (requiring coordination of eyes-heads-hand and increasing demands on attention and postural control) (Li, 2014). TCQ is a body–mind exercise, coordinating the movement of the limbs through lumbar winding movements (McAnulty et al., 2016). Therefore, TCQ may provide more stimulation for core muscles and improve the flexibility and stability of the spine. It may also enhance directly transferable skills in daily life, such as reaching and turning, going up/down stairs and walking.

##### Protocol

The eight movements have been demonstrated in our published article (R. Wang et al., 2022). The eight movements combine forward and backward steps, left and right steps and diagonal steps with rotating waist. The consistent exercise schedule includes three parameters: frequency (three times a week), time (60 min per session) and content (10 min of warm-up, eight core forms and 5 min cool-down). Method of inhaling and exhaling will be integrated into the TCQ forms. During the initial 3 weeks, classes will focus on learning these eight forms. At the fourth week, participants will learn to unite the forms. In the last 8 weeks, classes will concentrate on practice. To ensure the best curative effect, we conduct TCQ classes for participants with qualified coaches who finished training delivered by senior TCQ professor before the intervention classes. Apart from a coach, each class is provided with an assistant to provide right action feedback to the participants.

##### Exercise Intensity Monitoring

Given that our participants are mainly elderly people, exercise intensity will be closely monitored throughout the study. During the learning phase, participants will be instructed to exercise at which their rating of perceived exertion (RPE) is characterized as being “light to moderate” (Scherr et al., 2013). In the last 8 weeks, the intensity progresses to “moderate” (equals to 4–6 scores). Exercise intensity will be assessed weekly by the class assistant. All participants need to record their weekly home exercise and adverse events on the given file after class (see Supplementary Additional File 1).

#### Physical Therapy Group

After physical examination and special lumbar tests, we will divide the group into four types (type 1: LBP with mobility deficits; type 2: LBP with movement control impairment; type 3: LBP with referred pain; type 4: LBP with radicular pain) according to the World Health Organization’s International Classification of Functioning, Disability, and Health (Delitto et al., 2012) and the American Physical Therapy Association (APTA) clinical guideline of LBP (George et al., 2021). Different types of participants receive different types of exercises, such as position adjustment, flexibility exercise, stability exercise, specific trunk muscle activation exercise, McKenzie directional repetitive technique, and neural mobilization (see Supplementary Additional File 2). These exercise recommendations are based on the latest APTA guideline of LBP (George et al., 2021). The same therapist is responsible for in-group exercise for the same type of participants. The whole process will be implemented by the licensed therapist who have finished the prescribed training on the classification assessment and intervention, and recorded in the Classified Assessment and Intervention Form (see Supplementary Additional File 3).

##### Protocol

The exercise schedule includes three parameters: frequency (three times a week), time (60 min per session) and content (10 min of evaluation and warm-up, 40 min of therapist-directed exercise therapy, and 10 min of relaxation). Natural breathing is recommended during exercise.

##### Exercise Intensity Monitoring

For a more clinical setting, the 40 min of exercise intensity is controlled by the therapist’s evaluation of the participant. According to RPE, moderate intensity aerobic exercise equals to 4–6 scores of the modified Borg Scale. Participants shall record their weekly home exercise and adverse events on given file (see Supplementary Additional File 4).

### Specific Notes

Participants accept intervention after baseline data collection. For 12 weeks, both groups receive supervised exercise, keep normal lifestyle and is not subjected to additional CLBP treatment. All participants are able to keep their routine medical visits with physicians. The research staff records any changes made to treatment but do not change or recommend changes in medical therapy. Participants will receive 50 yuan after the baseline assessment, 100 yuan for the final assessment, and, respectively, 50 yuan for two follow-up visits. Ensure the attendance of each participant for 3-month intervention through sign-in and telephone supervision. Participants with an attendance of 80% or above will be rewarded with an extra 100 yuan. Some participants may be absent on some weekdays, so we will plan to take a make-up session on weekends based on the number of participants to ensure the optimal attendance.

## Data Collection and Outcome Measurements

The outcomes and measurement timepoints are presented in Table 1. A demographic questionnaire which consists of participants’ characteristics (sex, age, body mass index, education background, and so on) and history of LBP will be completed before the intervention.

### Primary Outcome

#### Numerical Rating Scale–the Maximum and the Average Pain

We will use the NRS to measure the maximum and average pain intensity at 12 weeks. The NRS is an 11-point (from 0 to 10) scale, and a score of ‘0’ or ‘10’ means no pain or unbearable pain, respectively (Williamson and Hoggart, 2005; Maughan and Lewis, 2010). Participants will assess their scores according to pain experience. The NRS has an excellent test-retest reliability with an intraclass correlation coefficient (ICC) of 0.92 (Chiarotto et al., 2019).

#### Roland Morris Disability Questionnaire–Back-Related Function

Roland Morris Disability questionnaire is suitable for evaluating the short-term changes of back-related function after intervention. The questionnaire consists of 24 questions, involving eight facets of walking, standing, bending, lying, dressing, sleeping, self-care, and daily activities (Roland and Morris, 1983). ‘Yes’ (1 point) or ‘No’ (0 point) is answered to each question, and a high total score equates to poor back function. A reduction of 30% on RMDQ score from baseline is identified as minimal clinically important difference for CLBP (Jordan et al., 2006). The RMDQ has an excellent test-retest reliability with an ICC of 0.91 (Brouwer et al., 2004).

### Secondary Outcomes

#### Lumbar Quantitative Sensory Testing

Pain threshold is a predicator of chronic pain and can be used to assess muscle sensitivity (Fischer, 1987). Patients with CLBP had lower pressure pain thresholds than healthy people (Farasyn and Meeusen, 2005; Giesbrecht and Battié, 2005). We will use a hand-held digital manometer (FDX 25 Digital Force Gage, 100× 0.1N) to evaluate pressure pain thresholds of lumbar and lower extremity muscles in elderly people with CLBP. And also, we will use Pathway sensory assessment system (Medoc Ltd., Israel) to evaluate hot pain thresholds and cold pain thresholds.

#### 4-Stage Balance Test

The 4-stage balance test is recommended by Disease Control and Prevention to assess static balance in the elderly (CDC, 2017). Participants start in sequence with standing with feet side-by-side, then standing with half step (he instep of one foot touches the toe of the other foot), standing in tandem stance (the heel of one foot touches the toe of the other foot), and standing on one foot. Participants try to hold each position without any support for 10 s measured with stopwatch. If participants failed in one of the positions, the test will be terminated. A total time of less than 30 s indicates high risk of falling.

#### Cognitive Performance

Cognitive decline is an important concern with aging process. Each elderly participant is expected to finish cognitive tests. The Trial making tests (TMT) mainly focus on memory and executive function (Llinàs-Reglà et al., 2017). TMT-A is carried out by participants using a pen to connect random distributed numbers 1 to 25 in sequence, and TMT-B is carried out by connecting distributed letters A to L in sequence. Record the completion time of TMT-A and TMT-B, respectively. The Stroop color- word test is used to evaluate attention control, executive function, and working memory (Washburn, 2016; W. Wang et al., 2021). Participants will response to the color which is congruent, incongruent, or irrelevant with the random word meaning (Hyodo et al., 2012). Record the response time and accuracy.

#### 6-Min Walk Test

The 6-min walk test (6MWT) is in measuring the distance that a participant can quickly walk on a flat and hard surface in 6 min. The distance recorded in meters will reflect the functional exercise level of daily physical activities (Peppin et al., 2014).

#### Geriatric Depression Scale

The 15-item Chinese revision of the Geriatric Depression Scale (GDS) is suitable for assessing the level of elderly depression. According to the items on the scale, participants answer ‘yes’ or ‘no’ to describe their feelings in the last 2 weeks. The higher the score (closer to 15) is, the higher level of depression is (Park and Kwak, 2021). The GDS has an excellent internal consistency (Albiński et al., 2011).

#### Self-Rating Anxiety Scale

The Self-rating Anxiety Scale (SAS) is a self-reported scale, in which items tap affective and somatic symptoms. Each item of the scale is rated by participants according to their actual status within the past week, using a 4-point scale ranging from 1 (none or a little of the time) to 4 (most or all of the time) (Shimada et al., 2016). Higher score indicates greater severity of anxiety. The SAS has a satisfactory internal consistency with a Cronbach’s alpha of 0.83 (Dunstan and Scott, 2020).

#### Pain Catastrophizing Scale

The Pain Catastrophizing Scale (PCS) is designed to assess catastrophic thinking associated with pain among patients with chronic pain (Wheeler et al., 2019). Participants will self-report 13 items related to painful experiences and the answer will be rated on a 5-point Likert scale from 0 (not at all) to 4 (all the time). A total score above 30 indicates clinically relevant level of catastrophizing. The test-retest reliability is excellent with an ICC of 0.94 (Xu et al., 2015).

#### Tampa Scale for Kinesiophobia

The Tampa Scale for Kinesiophobia (TSK) is a 17-item self-report measure to assess pain-related fear and is used extensively in patients with persistent LBP (Roelofs et al., 2004). The scale is rated on a 4-ponit Likert scale from 1 (strongly disagree) to 4 (strongly agree). A high score indicates high level of the fear of activity or reinjury. The TSK has a good reliability with an ICC of 0.86 (Wei et al., 2015).

#### Pittsburgh Sleep Quality Index

The Pittsburgh Sleep Quality Index (PSQI) can be used in assessing sleep quality and disturbances. The index ranges from 0 to 21 and consists of 24 questions covering seven domains. The lower the index is, the better the sleep quality or the lower the disturbance in sleep is (Mollayeva et al., 2016). The PSQI has an excellent test-retest reliability with a correlation coefficient of 0.77 (Tsai et al., 2005).

#### Short-Form 12

The Short-Form 12 (SF-12) is a brief health-related quality of life questionnaire. The questionnaire measuring eight domains can be used in calculating physical and psychological scores (Ware and Sherbourne, 1992). It is widely used in community-based health surveys and outcome assessment of physical and mental illnesses. Low scores indicate poor health status. The SF-12 shows satisfactory internal consistency both in the physical component summary (Cronbach’s alpha = 0.81) and mental component summary (Cronbach’s alpha = 0.83) (Su and Wang, 2019).

#### General Perceived Self-Efficacy Scale

The scale has 10 items, each of which is scored in integers from 1 (completely incorrect) to 4 (completely correct) (Zotti et al., 2007). High scores indicate better confidence to handle various things. General Perceived Self-efficacy Scale (GSES) can be considered a predictive variable of outcomes and an outcome itself. We use it to reflect participants’ confidence in their capability to increase self-management of chronic pain and perform daily coping tasks. The GSES has an adequate test-retest reliability with a correlation coefficient of 0.75 (Wu et al., 2004).

#### Expenses for Healthcare Monthly

To analyze the cost-effectiveness of TCQ and PT, we will collect participants’ expenses for treating LBP or other diseases monthly since the start of the intervention. We expect that practicing TCQ will reduce the cost for treating chronic diseases and improve quality of life.

#### Global Perceived Effect

Global Perceived Effect (GPE) numerical rating scale consists of seven choices (from 1 to 7 points), each representing the overall improvement in the participants’ symptoms after they completed the trial (Kamper et al., 2010). A good improvement indicates a high score. It can be useful in understanding the strengths and shortcomings of outcome measures for research. The participants will be asked to rate the degree of improvement or aggravation in their conditions since the beginning of the intervention. The GPE has an excellent test-retest reliability with an ICC of 0.90 (Kamper et al., 2010).

We will record the number of classes the participants attend and the reasons for absence. In addition, adverse events, which are related to interventions and home exercise, will be recorded (see Supplementary Additional Files 1, 4 for details).

## Data Management and Analysis

### Data Management

After recruitment, the researchers will replace the participants’ names with numerical codes in order to protect their privacy. Their data will be collected at baseline and week 12, 26, and 52, respectively. Two professional data workers are responsible for entering their paper files into the same database and cross-checking them for data’s accuracy. Eventually, all paper files and encrypted electronic files will be kept and backed up by the researcher leader. These files will be maintained in storage for 3 years after completion of the study.

### Data Analysis

Intention-to-treat analysis will be performed regardless of adherence. Microsoft excel 2016 and IBM SPSS Statistics 20.0 (SPSS Inc., Chicago, IL, United States) will be used in data analysis. Continuous variables (e.g., age, body mass index, NRS score, and RMDQ score) will be represented as mean ± standard deviation and be compared using analysis of the independent samples *t*-test. We will use the chi-squared test to analyze the categorical variables (e.g., gender, marital status, smoking, and exercise habits) between the TCQ group and PT group. The baseline demographics characteristics differences between groups will be compared using analysis of *t*-test, variance (ANOVA) or chi-squared tests. If potential confounding factors including gender, age, body mass index, smoking, exercise habits, etc., are present, we will prespecified an adjusted analysis, by using multiple linear regression. At the same time, missing values at week 12, 26, and 52 will be imputed using the multiple imputation.

The co-primary outcomes are the measurement of minimal clinically important difference in NRS and RMDQ scores in two groups after the 12-week intervention, and the paired-sample *t*-test will be carried out for the comparison of the differences. For secondary outcomes, the difference of continuous variables between groups will be compared using the analysis of independent-samples *t*-test. Expenses for LBP or other disease will be explored for cost-benefit analysis. Participants’ global perceived effect, adherence, and health-related quality of life will be compare using appropriate regression. The Fisher exact test will be used to compare adverse events. For the follow-ups phase, two-way repeated-measures analysis of variance (group × time) will be carried out to assess the differences of the primary outcomes (NRS score and RMDQ score) between the two groups. In all analyses, a *P*-value of <0.05 will be considered statistically significant.

### Data Monitoring

The study will be supervised by the Ethics Committee of the Shanghai University of Sport, whose members did not have any conflict of interest with this study. The principal investigators have access to all results and make the final decision to terminate the study. And also, they will grant project members the right to disseminate the results of this trial by publishing papers.

## Discussion

Finding an effective and inexpensive treatment for CLBP is valuable. Compared with the conventional non-invasive intervention for CLBP, TCQ is convenient and can be performed indoors and outdoors and by a single person or groups. For people of all ages, TCQ has the dual benefits of physical and mental conditioning. In this project, we will conduct an RCT to compare the effectiveness of TCQ with that of PT for CLBP. The TCQ practice program consists of eight movements for enhancing spinal stability and muscle strength of the waist and will be taught by professional teachers. Classification assessment and grouped-PT practice program based on patients’ functional performance and pain symptoms is carried out by licensed therapists. The intervention duration is 12 weeks, and 40 weeks of follow-ups is performed for the observation of long-term efficacy.

The strengths of this protocol are as follows. First, it will be the first RCT to compare modified TCQ with grouped-PT in patients with CLBP. The results of this trial will complement research in TCQ for LBP. Second, the 12-week intervention period and 40-week follow-up will be helpful in comparing the short- and long-term benefits of TCQ and PT. Third, by analyzing the medical expenses of the participants, the study will investigate whether TCQ has the benefit of saving medical costs. In addition, the study with large sample size will provide robust evidence of whether TCQ, rather than PT, can be a simple, effective, inexpensive and durable alternative therapy for chronic diseases. However, several potential limitations have been observed. First, all participants will not be blinded, and the recruitment criteria are limited to participants aged 50–80 years and without severe CLBP. Thus, the findings of study may not be appropriate for other age groups.

In conclusion, the purpose of this study is to estimate the effectiveness of TCQ in relieving pain intensity and improving back-related function and cognitive function on the basis of comparison with PT. The findings of this study will provide substantial evidence for TCQ as an effect alternative treatment for elderly people with CLBP.

## Ethics Statement

This study protocol has been approved by the Ethics Committee of the Shanghai University of Sport, China (number: 102772019RT039). A written informed consent will be obtained from each study participant. Written informed consent was obtained from the individual(s) for the publication of any potentially identifiable images or data included in this article.

## Author Contributions

X-QW had substantial contributions to the conception of the study. X-QW, DZ, LW, JL, JZ, YJ, YS, and RW designed the randomized controlled trial. RW, YS, H-YH, Z-WD, L-MW, K-YZ, and SK conducted the research. RW wrote the original draft of the manuscript. X-QW and RW participated in the revision of the draft. SK as one of the co-authors agreed to publish her images presented in Supplementary Additional File 2. All authors read and approved the final submitted version.

## Conflict of Interest

The authors declare that the research was conducted in the absence of any commercial or financial relationships that could be construed as a potential conflict of interest.

## Publisher’s Note

All claims expressed in this article are solely those of the authors and do not necessarily represent those of their affiliated organizations, or those of the publisher, the editors and the reviewers. Any product that may be evaluated in this article, or claim that may be made by its manufacturer, is not guaranteed or endorsed by the publisher.
