# Supplementary material for: Tai Chi Quan Versus Physical Therapy on Pain and Cognitive Performance for Elderly People With Chronic Low Back Pain: Study Protocol for a Randomized Controlled Trial
Source: Front Aging Neurosci. 2022 Jun 16;14:900430. doi: 10.3389/fnagi.2022.900430 (PMC9243751; doi:10.3389/fnagi.2022.900430)
Supplement: Supplementary file 1 [file Table_1.DOCX]

**Additional file 1** **Home exercise and adverse event record in the Tai Chi Quan group**

| Items Date: ×××× |
| --- |
| Question 1: Did you practice Tai Chi Quan at home today? And how long? |
| □ No □ Yes, minutes |
| Question 2: Did you do any other exercise? Such as walking, dancing, swimming, etc. |
| □ No □ Yes, minutes |
| Question 3: Did you have any of the following problems because of practicing Tai Chi Quan? |
| □ No □ Joint pain □ Increased back pain □ Leg pain □ Sciatica □ Neck pain □ Abdominal pain □ Dizziness |
| Question 4: Did you have any of the following problems because of participating in other exercises? |
| □ No □ Joint pain □ Increased back pain □ Leg pain □ Sciatica □ Neck pain □ Abdominal pain □ Dizziness |
| Question 5: Any other accident or injury? |
| □ No □ Yes, please specify: |

**Additional file 2 Grouped-exercises in the physical therapy group**

| **Classification** | **Exercise strategies** | **Pictures (for example)** |
| --- | --- | --- |
| Low back pain with mobility deficits | - Lumbar flexibility exercises (including flexion, extension, and rotation) - Positions adjustment - Hip flexibility exercises | 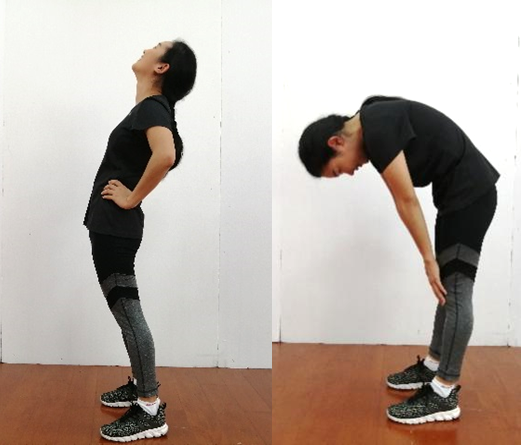  Flexion and extension  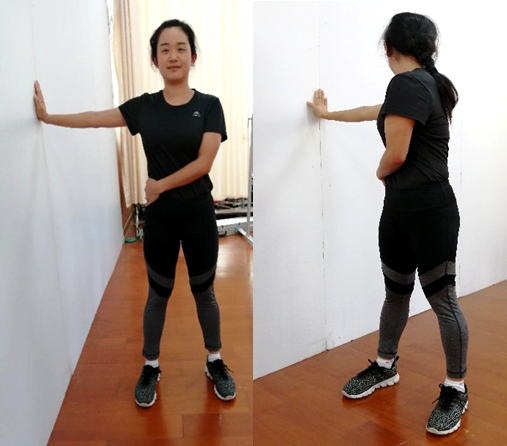  Rotation |
| Low back pain with movement control impairment | - Specific trunk muscle activation exercises (transversus abdominis and multifidus) - Core muscles stability training (including transversus abdominis, multifidus, longissimus thoracis, iliocostalis lumborum, quadratus lumborum, abdominal oblique, gluteus, etc.) | 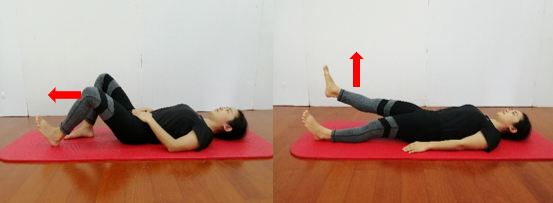  Transversus abdominis activation exercises  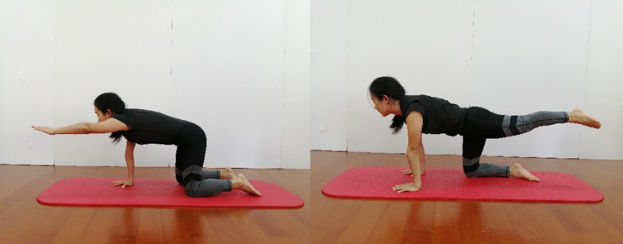  Position control  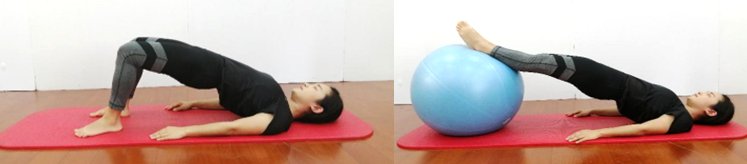  Hip bridge  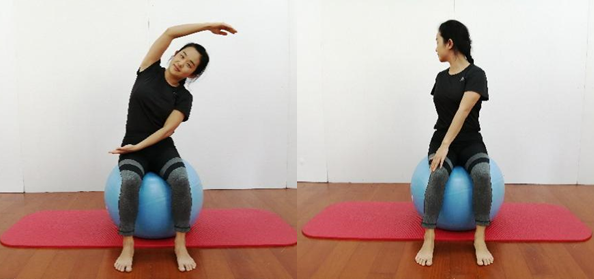  Sitting balance exercises |
| Low back pain with referred pain | - Positions adjustment - McKenzie directional repetitive technique | 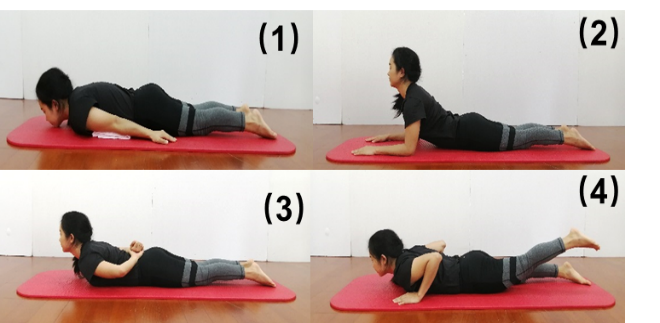  McKenzie technique with progressive exercises |
| Low back pain with radicular pain | - Stretching - Neural mobilization | 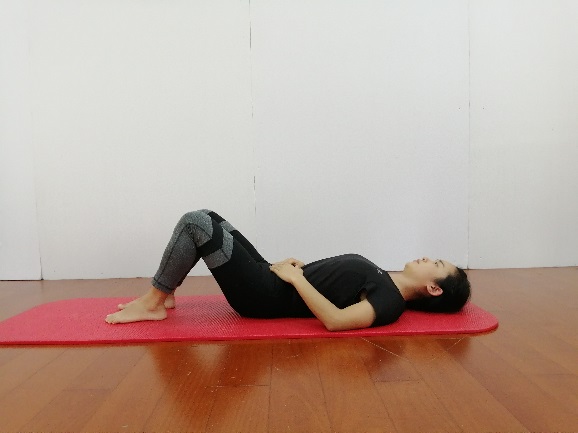  Pelvic rotation  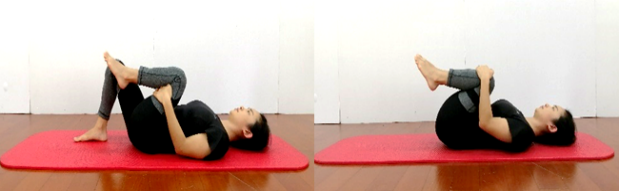  Gluteus stretching  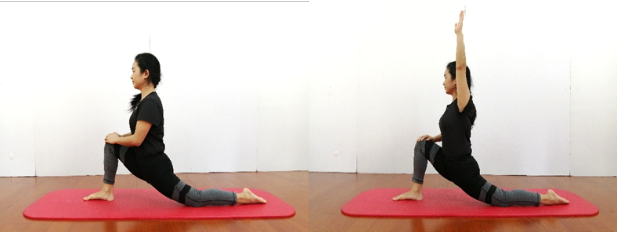  Hip extension  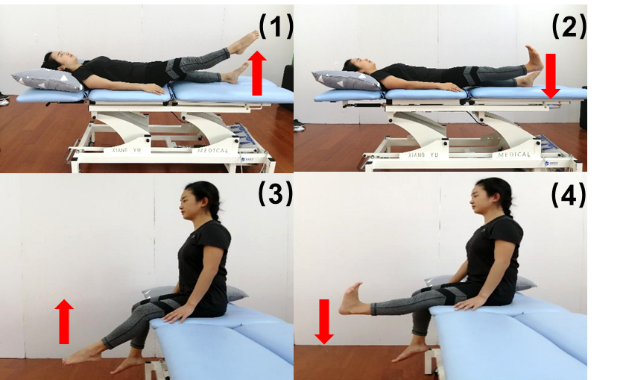  Neural mobilization |

**Additional file 3** **Classified assessment and intervention form of low back pain in the physical therapy group**

| Items | Contents |
| --- | --- |
| Therapist | Name: Evaluation date: |
| Participant | Name: Age: |
| Classification | □with mobility deficits □ with movement control impairment □with referred pain □ with radicular pain |
| Evidence of the evaluation | Physical examination and special lumbar tests |
| Rehabilitation recommendation | □ Position adjustment □ Flexibility exercise  □ Stability exercise □ Neural mobilization  □ McKenzie directional repetitive exercise □ Stretching  □ Specific trunk muscle activation exercise |
| Other notes | □Hypertension □Diabetes □Coronary heart disease  □Neck pain □Joint pain □Chronic lung disease □Other |

**Additional file 4** **Home exercise and adverse event record in the physical therapy group**

| Items Date: ×××× |
| --- |
| Question 1: Did you have any of the following problems because of physical therapy? |
| □ No □ Joint pain □ Increased back pain □ Leg pain □ Sciatica □ Neck pain □ Abdominal pain □ Dizziness |
| Question 2: Did you do any other exercise? Such as walking, dancing, swimming, etc. |
| □ No □ Yes, minutes |
| Question 3: Did you have any of the following problems because of participating in other exercises? |
| □ No □ Joint pain □ Increased back pain □ Leg pain □ Sciatica □ Neck pain □ Abdominal pain □ Dizziness |
| Question 4: Any other accident or injury? |
| □ No □ Yes, please specify: |
